# Fukushimura Brain Bank ‐Nursing home based brainbank project in Japan‐

**DOI:** 10.1002/alz70855_105293

**Published:** 2025-12-24

**Authors:** Daita Kaneda, Norihiro Ogawa, Takeshi Kanesaka, Chie Hishida, Keita Sakurai, Hiroyasu Akatsu, Hashizume Yoshio

**Affiliations:** ^1^ Institute of Neuropathology, Fukushimura Hospital, Toyohashi, Japan; ^2^ National Center for Geriatrics and Gerontology, Obu, Japan; ^3^ NGCC, obu, Japan

## Abstract

**Background:**

The Fukushimura (welfare village), located in Toyohashi city, Japan, is a unique complex of various nursing home facilities including dementia homes, Day‐care houses, homes for disabled and mentally retarded, and the Fukushimura Hospital. This village is totally managed by private sector, the Sawarabi Medical Cooperative. About 800 elderly people reside in this area. The hospital plays a pivotal role within the village and acts as the central facility for each nursing homes. Fukushimura BrainBank (FBB) was established in 1990. The neuropathological investigation of FBB was initially conducted by Kenji Kosaka, who initially reported cortical Lewy bodies. Our research institute was approved in 2004 by the Ministry of Education, Culture, Sports, Science and Technology to apply for governmental grants.

**Method:**

Advance directives for brain donation have been announced. Residents receive regular medical care at the hospital. Detailed serial imaging tests including MPRAGE T1 volume datas and diffusion tensor imaging (DTI) using 1.5T MRI (Siemens) are obtained in the clinical setting. Clinical datas including behavioral and psychological symptoms are collected from medical records and each nursing homes at the autopsy. Donation of retina is an optional offer at the autopsy.

For collecting CNS specimens, the brains are separated into two hemispheres, one for neuropathological analyses with using several antibodies for beta‐amyloid, phosphorylated tau, alpha‐ synuclein, and TDP‐43. Modified gallyas‐silver staining is applied for evaluating argyrophilic grain disease. The other hemispheres are stored for frozen sections.

**Result:**

At the end of 2024, 864 of frozen brains with detailed medical record are stored in FBB and readily available for research. 68brains with DTI scans are collected. The number of consents for retinal donation became 158, which is expected as one of the largest Asian retinal tissue bank of post‐mortem. We already have over 20 collaborators and supply our samples for more than 30 research institutes in Japan.

**Conclusion:**

The challenge of FBB will play a unique and important role to clarify cliniconeuropathological correlation of the nursing home residential with various geriatric conditions.

